# From Food to Mood: Psychological and Psychiatric Impact of Diet in Bipolar Disorder

**DOI:** 10.3390/nu17233728

**Published:** 2025-11-27

**Authors:** Giuseppe Marano, Gianluca Boggio, Francesca Abate, Emanuele Caroppo, Gianandrea Traversi, Osvaldo Mazza, Esmeralda Capristo, Eleonora Gaetani, Marianna Mazza

**Affiliations:** 1 Unit of Psychiatry, Fondazione Policlinico Universitario A. Gemelli IRCCS, Largo Agostino Gemelli 8, 00168 Rome, Italy; 2 Department of Neurosciences, Università Cattolica del Sacro Cuore, 00168 Rome, Italy; 3 Department of Mental Health, Local Health Authority ASL Roma 2, 00159 Rome, Italy; emanuele.caroppo@aslroma2.it; 4 Unit of Medical Genetics, Department of Laboratory Medicine, Ospedale Isola Tiberina-Gemelli Isola, 00186 Rome, Italy; gianandrea.traversi@gmail.com; 5 Spine Surgery Department, Bambino Gesù Children’s Hospital IRCCS, 00168 Rome, Italy; 6 Department of Translational Medicine and Surgery, Fondazione Policlinico Universitario A. Gemelli IRCCS, Università Cattolica del Sacro Cuore, 00168 Rome, Italy; esmeralda.capristo@policlinicogemelli.it (E.C.);; 7 Department of Medical and Surgical Sciences, Fondazione Policlinico Universitario A. Gemelli IRCCS, 00168 Rome, Italy; 8 Unit of Internal Medicine, Cristo Re Hospital, 00167 Rome, Italy

**Keywords:** bipolar disorder, nutritional psychiatry, diet, mood, mental health

## Abstract

Bipolar disorder (BD) is a severe psychiatric illness characterized by recurrent mood episodes and significant psychosocial impairment. Emerging evidence supports a bidirectional link between diet and mental health, with growing interest in nutritional psychiatry. This narrative review examines the psychological and psychiatric impact of diet in BD, focusing on biological mechanisms (gut–brain axis, neuroinflammation, oxidative stress, neurotransmitter synthesis, and HPA axis dysregulation) and the role of specific dietary patterns, including Western, Mediterranean, ketogenic, and anti-inflammatory diets. Key micronutrients such as omega-3 fatty acids, B-vitamins, magnesium, and vitamin D are explored in relation to mood regulation. This review also addresses psychological factors, including emotional eating, disordered eating behaviors, and the symbolic meaning of food in BD. Furthermore, it highlights the integration of nutritional psychoeducation into psychotherapy, the impact of comorbidities (e.g., obesity, metabolic syndrome), and the role of lifestyle factors such as sleep and physical activity. Despite promising findings, current research is limited by methodological heterogeneity. Future perspectives should include interdisciplinary, personalized interventions that incorporate nutritional strategies into standard care for BD.

## 1. Introduction

Bipolar disorder (BD) is a long-lasting and recurring mental health condition that impacts about 1–2% of people worldwide, making it one of the top causes of disability among young adults [1]. It is marked by shifts between manic, hypomanic, and depressive episodes, leading to significant challenges in daily life, a heightened risk of suicide, and a shorter life expectancy [2,3]. There is growing evidence that BD it affects multiple systems in the body, including immune, hormonal, and metabolic functions. Many patients also experience lifestyle changes [4], like poor sleep, lack of exercise, and unhealthy eating habits that can make their condition worse [5]. In recent years, the field of nutritional psychiatry has gained increasing attention, looking into how our diet and specific nutrients can impact brain function, mood, and how well treatments work [6,7].

It is becoming clear that what we eat plays a crucial role in influencing oxidative stress, inflammation in the brain, how our mitochondria function, and even the makeup of our gut bacteria, all of which are important in understanding BD. Diets that are heavy in processed foods, sugars, and saturated fats, like the typical Western diet, have been linked to increased inflammation, oxidative damage, and higher rates of metabolic issues in those with BD [8,9]. Conversely, eating patterns that are rich in anti-inflammatory and nutrient-dense foods, such as the Mediterranean diet, have been associated with better mood stability, lower levels of depression, and improved cognitive function [10].

From a biological perspective, various nutritional elements play a role in the neural and endocrine pathways that are important for BD. One key player is the gut–brain axis, which serves as a crucial two-way communication link between our gut microbiota and the central nervous system [11].

Research has shown that changes in the diversity of gut microbes can be seen in individuals with BD, potentially affecting emotional regulation through immune responses and the production of neuroactive substances [12]. On a similar note, imbalances in inflammation and oxidative stress can lead to neuronal damage, mitochondrial issues, and hindered neuroplasticity [13]. Thankfully, dietary antioxidants, polyphenols, and omega-3 fatty acids can help protect against these harmful processes [14,15].

Micronutrients like folate, vitamin B12, zinc, magnesium, and vitamin D are vital for synthesizing neurotransmitters and maintaining the integrity of myelin [16,17]. The lack of these nutrients can lead to cognitive decline, fatigue, and a higher risk of experiencing depressive episodes. Among these nutrients, omega-3 polyunsaturated fatty acids (PUFAs) are especially important for keeping neuronal membranes flexible and supporting serotonin transmission. Clinical studies indicate that taking supplements like eicosapentaenoic acid (EPA) and docosahexaenoic acid (DHA) might help alleviate depressive symptoms and enhance overall functioning in patients with BD, although the results can vary [18,19]. In BD, it is important not only to consider the biological side; eating habits also play a big role, shaped by psychological and sociocultural factors. Things like emotional eating, binge-eating tendencies, and strict dietary rules often pop up as unhealthy ways to cope with emotional ups and downs [20,21]. Food can take on a deeper meaning, serving as a way to manage emotions and rebuild identity during tough times. This highlights why it is crucial to combine nutritional strategies with therapy to help stabilize mood over the long haul. Lifestyle choices like exercise, sleep patterns, and even substance use work together with diet to impact the course of the illness [22]. Issues like obesity, metabolic syndrome, and weight gain from antipsychotic medications make things even trickier, intertwining our physical and mental health journeys. Looking at nutrition through a comprehensive biopsychosocial lens for BD could offer both preventive and therapeutic advantages. Even though there are some encouraging findings, the research is often hampered by small sample sizes, varied methods, and a lack of consistent dietary evaluations [23]. We need more interdisciplinary studies to better understand the connections and find biomarkers that link our metabolism, gut health, and inflammation to mood regulation [23]. Nutritional psychiatry is an exciting new area in personalized medicine, pushing for the inclusion of dietitians and lifestyle experts in psychiatric care teams. This review seeks to provide a comprehensive overview of the current evidence regarding the psychological and psychiatric effects of diet on BD. It emphasizes the biological mechanisms at play, various dietary patterns, the importance of micronutrients, and eating behaviors. Additionally, it explores the clinical implications and suggests future pathways for developing personalized, patient-centered nutritional interventions for individuals with BD. This manuscript is based on a narrative review framework. Literature was identified through targeted searches in PubMed, Scopus, and Google Scholar using terms related to “bipolar disorder”, “diet”, “nutrition”, “inflammation”, “microbiota”, “micronutrients”, and “eating behaviors”. Studies were selected according to four criteria: (1) direct relevance to bipolar disorder; (2) contribution to biological, psychological, or clinical mechanisms; (3) methodological quality; and (4) recency. When evidence from BD-specific studies was limited, findings from major depressive disorder, general severe mental illness, or metabolic populations were included and are explicitly noted as extrapolations in the revised manuscript.

## 2. Nutritional Psychiatry: Mechanisms and Pathways

Recent breakthroughs in nutritional psychiatry have shed light on several biological pathways that show how our diet can impact brain function and mood regulation, especially in BD. These pathways include the modulation of the gut–brain axis, management of neuroinflammation and oxidative stress, and the provision of essential micronutrients that are crucial for neurotransmitter synthesis and neuroplasticity. Additionally, changes in the hypothalamic–pituitary–adrenal (HPA) axis and metabolic balance create another connection between nutritional factors and mood swings.

### 2.1. Gut–Brain Axis

The gut–brain axis is a fascinating and intricate communication network that links our gastrointestinal system with the central nervous system. This connection happens through various pathways, including neural, immune, and endocrine routes. It plays a crucial role in regulating digestion, managing stress responses, and processing emotions, and it is gaining recognition as a key player in mental health [20]. In the case of BD, research has pointed to gut dysbiosis as a potential indicator of disease activity [21]. This condition is marked by a decrease in microbial diversity and changes in the levels of important bacteria like *Faecalibacterium prausnitzii*, Lactobacillus, and Bifidobacterium. These beneficial bacteria produce short-chain fatty acids (SCFAs), such as butyrate, which help maintain the integrity of the intestinal barrier and offer anti-inflammatory and neuroprotective benefits. Some microbiome findings originate from studies in healthy volunteers or depressive samples and are therefore used as mechanistic extrapolations to BD.

Higher levels of pro-inflammatory bacteria like Flavonifractor and lower levels of Christensenellaceae have been associated with oxidative stress, low-grade inflammation, and more severe symptoms in individuals with BD. Clinical studies have shown that probiotics containing Lactobacillus and Bifidobacterium can help alleviate depressive and cognitive symptoms while also reducing the risk of relapse in patients with stable BD [22]. Additionally, diets that are rich in fiber, polyphenols, and fermented foods, like the Mediterranean diet, promote microbial diversity and bolster resilience against mood fluctuations [23].

### 2.2. Neuroinflammation and Oxidative Stress

Chronic inflammation and oxidative imbalance play a key role in the pathophysiology of BD, leading to neuronal damage, reduced neuroplasticity, and mood dysregulation [24]. Part of the mechanistic evidence on inflammation and oxidative stress derives from transdiagnostic or non-psychiatric samples and is extrapolated to BD due to shared biological pathways.

Research consistently shows elevated levels of IL-6, TNF-α, and CRP during both manic and depressive episodes [25]. The activation of microglia, which shifts towards a pro-inflammatory M1 phenotype, causes mitochondrial dysfunction and cell death. If the anti-inflammatory M2 state is not restored, it can lead to neurodegeneration [26].

Frequent mood episodes result in allostatic overload, which heightens oxidative stress and speeds up neuroprogression [27]. Diet has a significant impact on these processes: a high intake of saturated fats and refined sugars can trigger low-grade systemic inflammation, while omega-3 fatty acids, antioxidants, and vitamins offer protective benefits. Studies have shown that supplementing with EPA and DHA can help reduce pro-inflammatory cytokines and alleviate depressive symptoms, especially when combined with traditional medications [28]. This relationship between dietary patterns and inflammation is not unique to psychiatric disorders but reflects broader systemic effects. For instance, Kupczyk et al. synthesized evidence showing that dietary interventions significantly modulate inflammatory and oxidative pathways in chronic autoimmune arthritis, underscoring the transdiagnostic relevance of diet–inflammation mechanisms and supporting the plausibility of similar effects in BD [29].

### 2.3. Neurotransmitter Synthesis and Micronutrients

Mood regulation hinges on the proper synthesis of neurotransmitters, especially serotonin, dopamine, and GABA, which in turn relies on dietary precursors and enzymatic cofactors [30]. Amino acids like tryptophan and tyrosine are crucial for producing serotonin and catecholamines, and their levels are affected by what we eat Micronutrients such as folate, vitamin B12, zinc, and magnesium play vital roles in neurotransmitter metabolism and maintaining neuronal health [17].

Folate and B12 are involved in one-carbon metabolism and help create S-adenosyl-methionine (SAM), a key player in producing serotonin and dopamine [31]. The lack of these vitamins can lead to higher homocysteine levels, increased oxidative stress, and depressive symptoms in BD [32]. Zinc and magnesium also help regulate NMDA and GABA receptors, which are important for maintaining the balance between excitatory and inhibitory signals in the brain and supporting neuroplasticity. Low levels of zinc and magnesium in the blood have been linked to more severe symptoms and cognitive issues, while supplementation has shown some promise in providing mild antidepressant effects [33].

### 2.4. Hypothalamic–Pituitary–Adrenal Axis and Metabolic Dysregulation

The HPA axis plays a crucial role in managing our stress response by regulating cortisol levels. When this system no longer functions correctly, which is often seen in BD, it can lead to higher baseline cortisol levels and a failure in feedback inhibition [34]. If the HPA axis is constantly activated, it can result in oxidative stress, shrinkage of the hippocampus, and various metabolic issues like insulin resistance and obesity [35].

**Figure 1:**
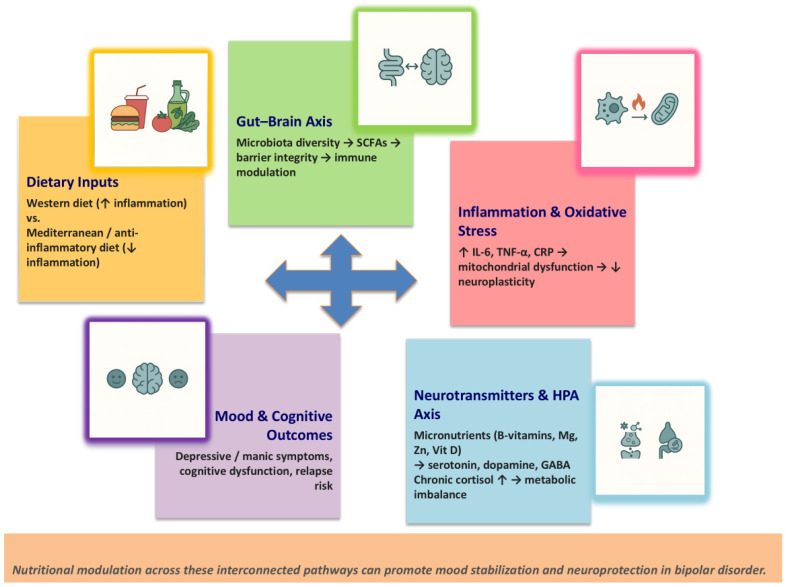
Biological pathways connecting diet and mood regulation in bipolar disorder. Note. Schematic representation of the biological pathways connecting diet and mood regulation in bipolar disorder. Nutritional factors modulate the gut–brain axis, inflammation, oxidative stress, neurotransmitter synthesis, and HPA axis activity, all contributing to emotional and metabolic homeostasis. Abbreviations: CRP, *C*-reactive protein; GABA, gamma-aminobutyric acid; HPA, hypothalamic–pituitary–adrenal; IL-6, interleukin-6; SCFAs, short-chain fatty acids; TNF-α, tumor necrosis factor-alpha; ↑, increase; ↓, decrease; →, induces.

What we eat and our overall metabolic health have a significant impact on the HPA axis. Diets high in fat and sugar can ramp up cortisol production and trigger systemic inflammation, while a diet rich in nutrients and anti-inflammatory foods can help restore a healthier neuroendocrine balance [35]. By combining nutritional strategies with medication and therapy, we might be able to lessen metabolic strain and promote better long-term stability [36]. The main biological mechanisms linking nutrition and mood regulation in BD are summarized in Figure 1. Nutritional factors modulate the gut–brain axis, inflammation, oxidative stress, neurotransmitter synthesis, and HPA axis activity, all contributing to emotional and metabolic homeostasis.

## 3. Dietary Patterns and Bipolar Disorder

Dietary habits are incredibly important when it comes to our physical and mental well-being. For those dealing with BD, certain eating patterns can really impact mood stability, inflammation, and how our metabolism works. It is vital to understand how various diets, like Western, Mediterranean, ketogenic, and plant-based, affect our brain and psychological health. This knowledge is key to creating effective treatment strategies that take a holistic approach.

### 3.1. Western vs. Mediterranean Diet

Dietary choices have a significant impact on the metabolic, inflammatory, and neurobiological processes that are involved in BD. Two contrasting dietary patterns, the Western and Mediterranean diets, showcase how different nutritional approaches can lead to vastly different outcomes for both mental and physical health [37]. The Western diet (WD) is known for its heavy reliance on ultra-processed foods, refined carbs, saturated and trans fats, red and processed meats, and sugary drinks, while lacking in fruits, vegetables, and whole grains [38]. This kind of eating can trigger systemic inflammation, insulin resistance, oxidative stress, and gut imbalances, all of which can exacerbate mood instability [39]. Research has shown that following a WD is linked to higher levels of *C*-reactive protein (CRP), IL-6, and TNF-α, which are biomarkers that tend to spike in BD patients during mood episodes [40].

The Mediterranean diet (MD) focuses on a rich variety of fruits, vegetables, legumes, nuts, whole grains, olive oil, and fish, all of which are packed with antioxidants, fiber, and omega-3 fatty acids [41]. The MD is known for its anti-inflammatory and neuroprotective benefits, as it helps balance the gut microbiome, improves insulin sensitivity, and supports both endothelial and mitochondrial health [42].

Observational studies reveal that individuals with BD often stick to the MD less than healthy individuals do, and a poorer diet quality is linked to larger waist sizes, increased insulin resistance, and more severe depressive symptoms [43]. In contrast, those who adhere closely to the MD tend to experience lower levels of systemic inflammation, better cognitive function, and a reduced risk of relapse [44]. It should be noted that part of this evidence derives from observational studies in general or depressive populations and is therefore partially extrapolated to BD.

### 3.2. Ketogenic and Low–Glycemic Index Diet

The ketogenic diet (KD) has been making waves as a possible complementary treatment for BD, thanks to its effects on mitochondrial metabolism, neuroinflammation, and neurotransmission [45]. Essentially, the KD is all about high fat, moderate protein, and low carbohydrates, which helps the body enter a state of ketosis. This shift encourages the body to burn fat for energy and produce ketones, leading to improved mitochondrial efficiency and lower oxidative stress [46]. Recent studies are hinting that the KD might help stabilize mood by influencing the transmission of glutamate and GABA, enhancing neuroplasticity, and normalizing the activity of the HPA axis.

Small open-label studies involving euthymic BD patients have shown promising results, with participants experiencing better mood stability, improved cognitive function, and even weight loss after sticking to the KD for 6 to 8 weeks [47]. On another note, low-glycemic index diets (LGID) that cut down on refined carbs and emphasize complex carbohydrates have been associated with better blood sugar control and fewer mood swings [48]. Meals high in glycemic load can lead to spikes in glucose and insulin after eating, which might hurt cognitive performance and lead to feelings of fatigue, irritability, and anxiety symptoms that often overlap with depressive episodes in BD [49]. Controlled feeding studies in adults with overweight or metabolic abnormalities indicate that low–glycemic load diets can stabilize postprandial glucose and insulin responses, leading to better subjective mood and lower fatigue levels [50]. Most LGID data come from overweight or metabolically impaired samples, and these findings are extrapolated to BD due to shared metabolic pathways.

Considering that more than half of BD patients struggle with insulin resistance, metabolic strategies like KD and LGID could potentially enhance both mental and physical health outcomes [51]. However, sticking to these diets long-term and ensuring their safety are challenges that still need to be addressed in future research. It is important to emphasize that BD-specific evidence for the ketogenic diet remains preliminary and is based primarily on small open-label studies with short follow-up periods. Robust randomized controlled trials in BD populations are still lacking. In addition, clinicians should be aware of potential risks and contraindications associated with ketogenic dietary strategies, including gastrointestinal symptoms, dehydration, electrolyte imbalance, dyslipidemic changes, and the need for careful monitoring in individuals with metabolic or cardiovascular comorbidities. Given the complexity of BD and its frequent metabolic burden, implementation of ketogenic interventions should be individualized, supervised by clinicians familiar with the diet, and integrated into multidisciplinary care.

### 3.3. Plant-Based and Anti-Inflammatory Diets

Plant-based diets that are loaded with fruits, vegetables, legumes, and whole grains offer a wide range of bioactive compounds like polyphenols, flavonoids, carotenoids, and vitamins that help manage oxidative stress and inflammation [52]. These nutrients also play a role in shaping the gut microbiome and boosting the production of short-chain fatty acids, which can enhance both metabolic health and emotional well-being [53]. Diets that focus on anti-inflammatory foods, particularly those rich in omega-3s, plant polyphenols, and a balanced omega-6 to omega-3 ratio, have been linked to lower levels of CRP, IL-6, and TNF-α [54]. For individuals with BD, these dietary choices might help lessen oxidative stress, promote the expression of brain-derived neurotrophic factor (BDNF), and support the brain’s adaptability [55]. Recent clinical research indicates that BD patients who adopt vegetarian or mostly plant-based diets often experience fewer depressive symptoms, better psychosocial functioning, and improved metabolic indicators like BMI and lipid profiles [56].

Additionally, long-chain omega-3 fatty acids from fish or algae can further amplify anti-inflammatory benefits and may lower the risk of relapse [55]. Some of the evidence supporting anti-inflammatory dietary effects originates from non-BD populations (e.g., metabolic syndrome or general community samples) and is therefore indirectly applied to BD. On the other hand, strictly vegan or poorly balanced plant-based diets can result in deficiencies in essential nutrients like vitamin B12, iron, zinc, and omega-3 fatty acids, all of which are vital for neurotransmitter production and overall brain health [57]. For this reason, personalized nutritional planning, ideally combined with psychiatric support, is crucial for achieving the best outcomes. A comparative summary of the main dietary patterns investigated in BD and their biological and clinical implications is reported in Table 1.

## 4. Micronutrients and Mood Regulation

Micronutrients play a vital role as cofactors in the synthesis of neurotransmitters, energy metabolism, and maintaining oxidative balance. There is a growing body of evidence indicating that certain nutritional deficiencies may be linked to the development of BD, affecting how severe symptoms are, how well treatments work, and even cognitive abilities [58]. Among the nutrients that have been most thoroughly researched are omega-3 fatty acids, B vitamins, zinc, magnesium, iron, selenium, and vitamin D. Because micronutrient studies specific to BD remain limited, several findings presented in this section derive from major depressive disorder or general psychiatric populations and are therefore partial extrapolations.

Table 2 provides an integrated overview of key micronutrients implicated in bipolar disorder, summarizing their biological mechanisms, clinical evidence, and potential therapeutic implications.

### 4.1. Omega-3 Polyunsaturated Fatty Acids (PUFAs)

PUFAs, particularly eicosapentaenoic acid (EPA) and docosahexaenoic acid (DHA), play a crucial role as structural elements in neuronal membranes and help regulate inflammatory and serotonergic pathways [15]. They work by lowering the production of pro-inflammatory cytokines while boosting neuroplasticity and synaptic function. A lack of omega-3s is linked to an increase in depressive symptoms and cognitive issues in BD [6]. Clinical trials have demonstrated that taking omega-3 supplements (usually around 1–2 g/day of EPA or a combination of EPA and DHA) can help alleviate depressive symptoms, especially when paired with medication [59]. These positive effects seem to stem from a reduction in oxidative stress, better stability of neuronal membranes, and the modulation of dopamine transmission [60]. Although the results can be mixed, omega-3 supplementation is generally viewed as a promising, safe, and well-tolerated addition to the treatment plan for managing BD [10].

### 4.2. B-Vitamins (Folate, B12, B6)

B-complex vitamins play a crucial role in one-carbon metabolism and the creation of monoamine neurotransmitters. Folate (vitamin B9) and cobalamin (vitamin B12) serve as essential cofactors in producing S-adenosyl-methionine (SAM), which is a key methyl donor in the synthesis of serotonin and dopamine [17]. When there are deficiencies in folate or B12, it can disrupt methylation processes, raise homocysteine levels, and lead to issues like increased emotional instability, cognitive difficulties, and a poor response to antidepressants [61]. Research, including both cross-sectional and genetic studies, has found that individuals with BD often have lower levels of plasma folate and B12, especially among those with a family history of suicide or more severe depressive symptoms [62]. While supplementation can help bring homocysteine levels back to normal and alleviate some lingering depressive symptoms, the effects tend to be modest and vary from study to study [63].

### 4.3. Magnesium, Zinc, Iron, and Selenium

Magnesium and zinc play crucial roles in how our neurons fire and communicate with each other. Magnesium serves as a natural blocker for NMDA receptors, while zinc helps manage the transmission of GABA and glutamate signals [64].

When we do not get enough of these minerals, it can lead to higher oxidative stress, immune system issues, and problems with emotional regulation, especially in BD [67]. Studies have shown that low levels of zinc and magnesium in the blood are common during both depressive and manic episodes, and these deficiencies often correlate with the severity of the illness [65]. Some research suggests that supplementing with these minerals can have mild antidepressant and anxiety-reducing effects, likely by helping to normalize the activity of the HPA axis and boosting the expression of brain-derived neurotrophic factor (BDNF) [66]. Iron and selenium, while not as widely researched, are also essential for keeping our mitochondria functioning well and defending against oxidative damage. A lack of iron can hinder oxygen transport and the production of important neurotransmitters, while too much iron can lead to oxidative stress [68]. Selenium plays a key role in thyroid hormone metabolism and acts as a cofactor for glutathione peroxidase, which helps protect neurons from oxidative harm. Disruptions in these trace elements might also contribute to the cognitive decline and emotional instability often seen in BD [67].

### 4.4. Vitamin D

Vitamin D acts like a neurosteroid and plays a variety of roles in brain development, immune function, and neurotransmission [69]. Vitamin D receptors are present all over the cortex, hippocampus, and limbic system, areas that are key players in the pathophysiology of BD [70]. When there is a deficiency, it is often linked to symptoms of depression, cognitive issues, and sleep disturbances. While some studies have noted higher vitamin D levels during the early or manic phases of BD, the majority of evidence suggests that long-term deficiency can lead to mood swings and other metabolic problems [71].

Supplementing with vitamin D might help improve mood and regulate circadian rhythms, especially for those who start with low levels, but the results can vary widely and depend on the dosage [72]. In summary, vitamin D seems to influence inflammatory and neurotrophic pathways by regulating serotonin production, maintaining calcium balance, and reducing oxidative stress, which highlights its potential as a valuable addition to BD treatment strategies [73].

## 5. Eating Behaviors, Mood, and Identity in Bipolar Disorder

Altered eating habits are becoming more recognized as an important aspect of BD, highlighting both biological risks and psychological adjustments. Issues like emotional eating, disordered behaviors such as binge eating or orthorexia, and struggles with body image are quite common, leading to a lower quality of life and various metabolic issues [74]. In the context of BD, food takes on a deeper significance, playing a role in managing emotions and shaping identity, which is why it should be included in comprehensive treatment plans.

### 5.1. Emotional Eating and Mood Episodes

Emotional eating, where people turn to food as a way to cope with negative feelings instead of actual hunger, is especially common during the low points of BD. In fact, around 40% of those affected report episodes of emotional overeating, often craving sugary and fatty foods [75]. This behavior is tied to how our reward and stress systems are regulated, which includes changes in dopamine signaling and the HPA axis [76]. In contrast, during manic or hypomanic phases, eating habits can flip dramatically, leading to less food intake, erratic meal times, and a higher reliance on caffeine, alcohol, or stimulants. This can throw off our body’s natural rhythms and how we manage metabolism [77,78]. Neuroimaging studies have shown that BD patients exhibit increased activity in the amygdala and nucleus accumbens when they encounter food cues, indicating a shift in how they process rewards [79]. Additionally, imbalances in hormones like leptin, ghrelin, and cortisol further highlight the connection between mood swings and appetite control [80]. Emotional eating in BD is also strongly shaped by dysregulation of reward processing, stress sensitivity, and altered interoceptive awareness. Neuroimaging studies demonstrate heightened responsivity of the amygdala and nucleus accumbens to food-related cues in BD, indicating a maladaptive reinforcement cycle during negative affect states [79]. Additionally, hormonal fluctuations involving leptin, ghrelin, and cortisol contribute to impaired satiety signaling and increased drive for high-calorie foods [80]. These interactions position emotional eating as a psychological coping mechanism that emerges from the convergence of biological vulnerability and affective instability.

### 5.2. Disordered Eating Patterns

The connection between BD and eating disorders (EDs) is quite significant, with lifetime prevalence estimates falling between 10% and 30% [74]. Binge eating disorder (BED) stands out as the most common, closely linked to impulsivity and emotional instability [81]. Individuals dealing with both BD and BED tend to experience more intense depressive episodes, higher rates of suicidality, and increased obesity compared to those with BD alone [82].

Orthorexia nervosa, which involves an unhealthy fixation on “healthy” or “pure” eating, has been getting more attention lately. While it is not officially recognized in diagnostic manuals, traits of orthorexia are more frequently seen in people with BD, especially among those who exhibit obsessive-compulsive behaviors and rigid thinking [83]. Although these behaviors might seem beneficial at first glance, they can ultimately lead to malnutrition and social withdrawal.

### 5.3. The Psychological Meaning of Food

Food is more than just fuel bodies; it plays a significant role in how individuals with BD manage their emotions and reshape their identities [84]. Research shows that many patients turn to specific eating rituals as a way to regain a sense of control or stability during their emotional ups and downs. The choices they make about food often mirror their mood and how they see themselves and their bodies [85]. Cultural influences also add layers to the psychological significance of eating. In Mediterranean cultures, for instance, meals are more than just a time to eat; they act as social anchors that foster a sense of belonging and stability in relationships elements that can be thrown off balance during mood swings [41]. Beyond its metabolic implications, food often becomes a symbolic regulator of self-coherence and emotional stability in individuals with BD. Patients frequently use food rituals to manage internal chaos, reduce uncertainty, or regain a sense of continuity during mood fluctuations [85]. These behaviors may reflect deeper processes related to attachment, identity, and emotional regulation, as eating practices can serve as temporary anchors during periods of affective instability. Evidence also suggests that disordered eating patterns in BD are intertwined with emotional dysregulation and impulsivity [74], reinforcing the psychological significance of nutritional behavior.

When dietary guidelines ignore personal significance or cultural practices, it can make it much harder for individuals to stick to nutritional or therapeutic plans [86].

### 5.4. Body Image and Self-Perception

Obesity is almost twice as common in BD compared to the general population, and it can lead to stigma, low self-esteem, and difficulties in sticking to treatment [87]. Weight gain can derive from the use of medications, especially atypical antipsychotics like olanzapine and clozapine, as well as mood stabilizers such as valproate [88]. There is a clear link between a higher body mass index (BMI) and issues with cognition and metabolism [89]. Notably, abdominal obesity has been tied to an increased risk of violent suicide attempts in those with BD, underscoring the intricate relationship between metabolic, psychological, and behavioral factors in this condition [90].

### 5.5. Clinical Interventions

Targeted interventions that focus on eating behaviors in binge disorders highlight the importance of teamwork among psychiatrists, psychotherapists, and dietitians [91]. By providing nutritional psychoeducation, it is possible to boost adherence and raise awareness about lifestyle choices. Meanwhile, cognitive–behavioral and dialectical behavior therapies play a crucial role in curbing binge eating and managing emotional dysregulation [92]. Integrating mindfulness-based methods into BD treatment may bolster emotional and metabolic resilience by enhancing stress regulation, improving interoceptive awareness, and modulating neural and hormonal pathways [93]. Additionally, incorporating dietary counseling into psychiatric care can lessen metabolic issues and improve overall outcomes [94].

## 6. The Role of Nutrition in Psychological Therapy

Bringing nutritional principles into the realm of psychological and psychiatric treatment is becoming a key focus in managing BD. Our diet plays a significant role, not just in biological processes like inflammation and neurotransmission, but also in cognitive, emotional, and behavioral aspects that are crucial for sticking to therapy and achieving long-term recovery [95].

### 6.1. Nutritional Psychoeducation

Nutritional psychoeducation is at the heart of lifestyle-focused therapy for BD. Programs designed to teach patients about the connections between their diet, medication, and mood management have proven effective in enhancing understanding, adherence, and metabolic health [96]. These psychoeducational sessions typically cover topics like glycemic control, portion sizes, and the benefits of anti-inflammatory foods, such as omega-3-rich fish, fruits, and vegetables [97]. Group interventions that blend dietary and psychological education tend to lead to better weight management, fewer relapses, and overall improved functioning [98]. Involving family members or caregivers in this process can make lifestyle changes more sustainable and help reduce feelings of stigma [99]. Nutritional psychoeducation also plays an important psychological function by helping patients reinterpret food not merely as a source of comfort or emotional regulation but as an element of self-management and illness insight. Group-based lifestyle interventions combining nutritional education with psychological skills have shown improvements in weight control, mood stability, and illness awareness [96,98]. By fostering self-efficacy, psychoeducation directly addresses maladaptive coping patterns such as emotional overeating, thus bridging biological and psychological aspects of BD.

### 6.2. Integration into Psychotherapy

Diet can serve as both a target for behavior change and a meaningful aspect of psychotherapy. Approaches like cognitive–behavioral therapy (CBT) and dialectical behavior therapy (DBT) have been tailored to include nutrition-focused modules that tackle issues like emotional eating, impulsivity, and self-care practices. This integration allows patients to identify mood-related triggers for unhealthy eating habits and encourages the development of healthier coping strategies. Mindfulness-based practices, including mindful eating techniques, have shown potential in decreasing binge eating episodes and fostering body awareness in individuals with BD [100]. These methods improve sensitivity to internal cues and help break the automatic connection between emotional distress and food consumption. In psychodynamic and humanistic approaches, food-related themes can be examined as reflections of identity, attachment, and self-coherence. This reflective aspect promotes autonomy and empowerment, ultimately supporting a recovery-oriented approach to care [101]. Psychotherapeutic approaches further strengthen the psychological dimension of nutritional behavior. CBT and DBT modules targeting emotional eating, impulsivity, and distress-driven food choices help patients identify triggers and develop healthier coping strategies [92,100]. Mindfulness-based interventions improve interoceptive awareness and attenuate automatic eating responses linked to mood fluctuations [93]. Integrating these strategies into BD-focused psychotherapy therefore positions diet not just as a lifestyle variable but as a psychological domain essential to relapse prevention and functional recovery.

### 6.3. Motivation and Adherence

One of the biggest hurdles in managing BD is keeping up with both medication and behavioral therapies. Engaging with nutrition can really boost a person’s sense of self-efficacy and control, acting as a great starting point for diving into more complex treatment options [102]. When patients notice real changes in their energy levels, sleep quality, or mood from dietary adjustments, they tend to be more involved in their therapy and stick to their medication better [103]. Bringing dietitians into psychiatric teams means that eating habits, nutrient gaps, and personal lifestyle goals can be assessed on an individual basis [104]. This collaborative approach has shown to lessen metabolic issues and enhance the overall quality of life for those with BD.

## 7. Comorbidities and Lifestyle Factors in Bipolar Disorder

BD is increasingly being seen as a complex condition that affects multiple systems, with intricate interactions among psychiatric, metabolic, and lifestyle factors. It is common to find medical issues like obesity, metabolic syndrome, cardiovascular disease, and sleep problems in individuals with BD, and these can lead to poorer clinical outcomes, less effective treatment responses, and even higher mortality rates [105,106]. For this reason, lifestyle changes aimed at these areas are crucial as a complement to medication management.

### 7.1. Metabolic Syndrome and Obesity

Metabolic syndrome impacts nearly 50% of people with BD, which is two to three times more than what we see in the general population [81]. The key factors (central obesity, insulin resistance, dyslipidemia, and hypertension) are intimately linked to chronic low-grade inflammation and structural or functional decline in cognition. Moreover, medications such as olanzapine and clozapine, and mood stabilizers like valproate, contribute significantly to weight gain, insulin resistance, and derangements in metabolic homeostasis [107,108]. Many patients with BD struggle to maintain healthy eating habits and often show low adherence to the Mediterranean diet, a dietary pattern known for its protective metabolic and anti-inflammatory effects. Poor adherence has been linked to higher body mass index (BMI), insulin resistance, and worse metabolic outcomes in this population [97]. On the flip side, embracing anti-inflammatory diets that are rich in omega-3 fatty acids, polyphenols, and fiber has been shown to improve lipid levels and stabilize mood [109]. Incorporating lifestyle changes that include diet, exercise, and psychoeducation can enhance metabolic health, support weight loss, and boost overall well-being [98].

### 7.2. Sleep and Circadian Rhythms

Sleep and the regulation of our circadian rhythms are crucial when it comes to understanding the pathophysiology of BD. Irregular sleep–wake patterns, insomnia, and hypersomnia not only serve as symptoms but can also trigger mood episodes [110]. When circadian rhythms are disrupted, it can throw off our metabolic and hormonal balance, leading to issues like weight gain, insulin resistance, and neuroinflammation [77]. To help stabilize these rhythms and prevent relapses, chronotherapeutic interventions such as light therapy, interpersonal and social rhythm therapy (IPSRT), and behavioral sleep regulation have shown promising results [111]. Additionally, nutrients like tryptophan, magnesium, and vitamin D play a role in supporting melatonin production and circadian regulation, underscoring the important link between nutrition, sleep, and mood [112].

### 7.3. Physical Activity and Sedentary Behavior

Physical inactivity is a common issue for patients with BD, and it can lead to worse metabolic and mental health outcomes [113]. Engaging in regular aerobic and resistance training can significantly improve symptoms of depression, boost cognitive abilities, and enhance cardiovascular health, all while helping to lower systemic inflammation [114]. Additionally, exercise plays a role in increasing BDNF levels and promoting neuroplasticity, which aligns well with nutritional strategies that target similar brain pathways [115]. However, despite these advantages, many individuals face obstacles like fatigue, lack of motivation, and side effects from medications that can hinder their ability to stick with an exercise routine. To tackle this, creating personalized exercise plans that are introduced gradually, along with psychoeducation and nutritional guidance, can help ensure that these habits are maintained over the long haul [116].

### 7.4. Substance Use and Addictive Behaviors

Substance use disorders (SUDs) are some of the most prevalent comorbidities associated with BD, with lifetime prevalence rates nearing 60% [117]. Substances like alcohol, nicotine, and stimulants can worsen mood swings, disrupt sleep, and throw metabolic balance out of whack [118]. New research indicates that the quality of one’s diet can influence how substance use affects oxidative stress and inflammation [119]. By incorporating nutritional support, especially antioxidant and anti-inflammatory foods, into addiction treatment, we might reduce the risk of relapse and enhance cognitive recovery [120].

## 8. Gaps in Research and Future Directions

Even though there is a growing interest in nutritional psychiatry, the research looking into how diet affects BD is still new. A lot of the existing studies tend to use small sample sizes, varied methods, and cross-sectional designs, which makes it hard to draw clear conclusions. Most of the trials have been centered around major depressive disorder, with only a handful actually examining specific nutritional interventions for people with BD.

### 8.1. Methodological Limitations

Current evidence is limited by the variability in how dietary assessments are conducted, the lack of control for confounding factors like medication use and socioeconomic status, and the short duration of follow-up periods [121]. It is also worth noting that randomized controlled trials focusing on specific dietary patterns or micronutrient supplementation are quite rare and often lack sufficient power [122]. Additionally, biomarkers that indicate nutritional status, such as omega-3 indices, vitamin D levels, or microbiome composition, are reported inconsistently, which makes it difficult to replicate findings and conduct meta-analyses [123]. Another challenge is the absence of standardized diagnostic tools for assessing disordered eating behaviors in individuals with BD. Issues like emotional eating, orthorexia, and binge-related symptoms are frequently evaluated using vague or self-reported measures, which diminishes their reliability and makes it hard to compare results across different studies [124].

### 8.2. Need for Interdisciplinary and Personalized Approaches

The intricacies of BD call for a comprehensive approach that weaves together psychiatry, nutrition, endocrinology, and behavioral sciences. Looking ahead, research should shift from simple correlations to a more personalized nutritional psychiatry, customizing dietary strategies based on individual biological profiles, circadian rhythms, and the side effects of medications [36]. Precision medicine that incorporates genomics, metabolomics, and microbiome studies could be key in pinpointing who will benefit from specific diets and in understanding why some treatments do not work for everyone [125]. For instance, analyzing gut microbiota could help tailor probiotic or prebiotic treatments to boost cognitive and emotional well-being [126]. Collaborative care models that bring together psychiatrists, psychologists, and registered dietitians are likely to improve treatment adherence, metabolic health, and overall quality of life for those with BD [127].

### 8.3. Translational and Clinical Implications

Bringing nutrition-focused strategies into clinical psychiatry is not merely advisable; it needs solid guidelines, proper training, and support from institutions [121]. We could really benefit from weaving nutritional psychoeducation into psychotherapy and rehabilitation programs, highlighting the importance of self-management, anti-inflammatory diets, and lifestyle changes [128]. To truly understand the impact of dietary interventions, we need longitudinal cohort studies and multicenter randomized controlled trials (RCTs) to assess causality, sustainability, and cost-effectiveness [129]. In addition, using digital tools like apps and wearables could help us track dietary habits in real-time and see how they connect to mood changes [130]. In short, nutritional psychiatry in BD is an exciting area that is still growing. Future research should prioritize interdisciplinary, longitudinal, and mechanistically oriented studies to elucidate the overarching connections between experimental findings and their translation into clinical practice [36].

## 9. Conclusions

BD is a complex and multifaceted condition where biological, psychological, and lifestyle factors interact in dynamic ways throughout the illness. There is growing evidence highlighting the importance of diet as both a biological influencer and a behavioral factor in maintaining mood stability. What we eat can affect neuroinflammation, oxidative stress, neurotransmission, and our circadian rhythms, all of which play a key role in the pathophysiology of BD. Integrating nutritional psychiatry into traditional treatment approaches marks a significant shift towards more holistic and patient-focused care. Dietary choices, especially those that focus on anti-inflammatory foods, Mediterranean-style eating, sufficient omega-3 intake, and a balanced array of micronutrients, can complement medication strategies and enhance long-term outcomes.

Translating these findings into clinical practice is still a challenge due to varying methodologies and the lack of standardized protocols. It is pivotal for psychiatrists, dietitians, and psychologists to work together to create evidence-based guidelines and scalable interventions. Ongoing studies should focus on understanding the intricate, two-way relationship between diet and mood regulation through mechanistic, longitudinal, and personalized studies. By merging molecular insights with digital tracking and educational frameworks, we could elevate nutritional psychiatry from a supplementary approach to a fundamental aspect of precision mental health. Ultimately, the aim is to reshape how we treat BD by adopting integrative, lifestyle-focused models that empower patients and enhance both their mental and physical well-being.

## Figures and Tables

**Table 1:** Main dietary patterns and their effects in bipolar disorder.

Dietary Pattern	Core Characteristics	Primary Biological Mechanisms	Observed or Proposed Effects in BD	BD-Specific vs. Extrapolated Evidence	References
Western diet	High in ultra-processed foods, refined carbohydrates, saturated and trans fats; low in fiber and antioxidants.	↑ Systemic inflammation, ↑ oxidative stress, gut dysbiosis, insulin resistance.	Associated with higher CRP, IL-6, TNF-α; greater mood instability, metabolic comorbidities, and poorer cognitive outcomes.	Extrapolated (general population, MDD, metabolic studies)	[38,39,40]
Mediterranean Diet	Rich in fruits, vegetables, legumes, nuts, olive oil, and fish; low in red and processed meat.	↓ Neuroinflammation and oxidative stress; improved mitochondrial and endothelial function; modulation of gut microbiota.	Linked to lower depressive symptoms, improved cognition, reduced relapse risk, and better metabolic profile.	Partially BD-specific (some BD data) + extrapolated evidence	[41,42,43,44]
Ketogenic Diet	High-fat, moderate-protein, low-carbohydrate; induces nutritional ketosis.	↑ Mitochondrial efficiency; ↓ oxidative stress; modulation of GABA/glutamate balance and HPA axis.	Improved mood stability, cognition, and weight control in small open-label studies; long-term safety yet to be established.	BD-specific (small open-label trials)	[45,46,47]
Low-Glycemic Index Diet	Focuses on complex carbohydrates, low refined sugars, and stable postprandial glucose.	Stabilization of insulin and glucose metabolism; reduced oxidative and inflammatory load.	Associated with fewer mood fluctuations, improved fatigue and energy levels, particularly in insulin-resistant patients.	Extrapolated (metabolic/overweight samples)	[48,49,50]
Plant-Based/Anti-Inflammatory Diets	Emphasis on fruits, vegetables, legumes, whole grains, polyphenol-rich and omega-3-rich foods.	Antioxidant and anti-inflammatory action; modulation of gut microbiota; ↑ BDNF expression.	Linked to lower depressive symptoms, improved metabolic indicators (BMI, lipids), and enhanced psychosocial functioning.	Partially BD-specific (few studies) + extrapolated evidence	[52,53,54,55,56]

Abbreviations: BD, bipolar disorder; MDD, Major Depressive Disorder; BDNF, brain-derived neurotrophic factor; CRP, *C*-reactive protein; HPA, hypothalamic–pituitary–adrenal; IL-6, interleukin-6; TNF-α, tumor necrosis factor-alpha; ↑, increase; ↓, decrease.

**Table 2:** Key micronutrients in bipolar disorder: mechanisms, evidence, and clinical implications.

Micronutrient	Primary Biological Mechanisms	Key Findings/Evidence in BD	References
Omega-3 fatty acids (EPA, DHA)	Anti-inflammatory effects; modulation of serotonergic and dopaminergic transmission; stabilization of neuronal membranes; reduction in oxidative stress	Lower omega-3 levels associated with greater depressive symptoms; supplementation (1–2 g/day) improves residual depressive symptoms and reduces inflammatory cytokines; beneficial as adjunct to medication	[6,10,15,18,19,28,29,59,60]
B-vitamins (Folate, B12, B6)	One-carbon metabolism; synthesis of SAM; monoamine neurotransmitter production; methylation processes	BD patients frequently show lower folate/B12 levels; deficiencies linked to cognitive symptoms, emotional instability, elevated homocysteine, and poorer antidepressant response; supplementation normalizes homocysteine	[17,31,61,62,63]
Magnesium	NMDA receptor regulation; modulation of excitatory/inhibitory balance; anti-inflammatory and antioxidant roles	Low serum magnesium during manic/depressive episodes; deficiency associated with increased severity and impaired stress regulation; supplementation shows mild antidepressant effects	[64,65,66]
Zinc	Regulation of GABA and glutamate transmission; antioxidant activity; involvement in neuroplasticity	Lower zinc levels associated with worse mood symptoms and cognitive impairment; some BD patients show elevated zinc under stable conditions; supplementation may exert antidepressant-like effects	[33,65,66,67]
Iron	Neurotransmitter synthesis; mitochondrial respiration; oxygen transport	Both deficiency and iron overload impact oxidative stress and neuroprogression; abnormalities may contribute to fatigue, cognitive dysfunction, and mood instability	[67,68]
Selenium	Cofactor for glutathione peroxidase; modulation of thyroid metabolism; antioxidant and anti-inflammatory pathways	Lower selenium associated with impaired antioxidant defense in BD; abnormalities linked to treatment with lithium or valproate; possible contribution to cognitive and emotional dysregulation	[67]
Vitamin D	Neurosteroid activity; immune regulation; serotonin synthesis; calcium homeostasis; circadian rhythm modulation	Low vitamin D linked to depressive symptoms, cognitive impairment, and sleep disturbances; supplementation beneficial mainly in deficiency states; abnormalities reported across mood phases	[69,70,71,72,73]

Abbreviations: BD, bipolar disorder; EPA, eicosapentaenoic acid; DHA, docosahexaenoic acid; SAM, S-adenosyl-methionine; NMDA, *N*-methyl-D-aspartate; GABA, gamma-aminobutyric acid.

## Data Availability

No new data were created or analyzed in this study.
